# Deep learning-based automated and universal bubble detection and mask extraction in complex two-phase flows

**DOI:** 10.1038/s41598-021-88334-0

**Published:** 2021-04-26

**Authors:** Yewon Kim, Hyungmin Park

**Affiliations:** 1 Department of Mechanical Engineering, Seoul National University, Seoul, 08826 Korea; 2 Institute of Advanced Machines and Design, Seoul National University, Seoul, 08826 Korea

**Keywords:** Mechanical engineering, Fluid dynamics

## Abstract

While investigating multiphase flows experimentally, the spatiotemporal variation in the interfacial shape between different phases must be measured to analyze the transport phenomena. For this, numerous image processing techniques have been proposed, showing good performance. However, they require trial-and-error optimization of thresholding parameters, which are not universal for all experimental conditions; thus, their accuracy is highly dependent on human experience, and the overall processing cost is high. Motivated by the remarkable improvements in deep learning-based image processing, we trained the Mask R-CNN to develop an automated bubble detection and mask extraction tool that works universally in gas–liquid two-phase flows. The training dataset was rigorously optimized to improve the model performance and delay overfitting with a finite amount of data. The range of detectable bubble size (particularly smaller bubbles) could be extended using a customized weighted loss function. Validation with different bubbly flows yields promising results, with AP_50_ reaching 98%. Even while testing with bubble-swarm flows not included in the training set, the model detects more than 95% of the bubbles, which is equivalent or superior to conventional image processing methods. The pure processing speed for mask extraction is more than twice as fast as conventional approaches, even without counting the time required for tedious threshold parameter tuning. The present bubble detection and mask extraction tool is available online (https://github.com/ywflow/BubMask).

## Introduction

Measurement techniques based on optical visualization are ubiquitous approaches that are now being adopted in the experimental investigations of diverse problems from biological (small scale) to industrial (large scale) phenomena^1–4^. This is also true while studying multiphase flows, where the simultaneous measurement of individual phases over a large region of interest, without disturbing the flow, is advantageous (compared to intrusive measurement methods) in understanding the interaction between phases. In dealing with a gas–liquid two-phase (bubbly) flow, in particular, it is critical to measure the spatiotemporal variation of the interfacial shape accurately for the purpose of analyzing the transport phenomena between phases^5–9^. While detecting bubbles using optical visualization, the major obstacle is to identify and track individual bubbles (and statistics including the size and velocity) from the overlapped bubble cluster. Numerous image processing techniques have been proposed as tools for effective bubble detection, such as the Hough transform^10,11^, breakpoint method^12,13^, and Watershed transform^9,14,15^. These methods have proved to be useful, but the application of a simple image processing filter is insufficient to process all images of bubbles with various geometrical features, because of the wide scatter of the flow conditions and optical settings of each study. Even in a single image of a bubbly flow, bubble images have different characteristics that cannot be readily distinguished using a single process (criteria). While addressing this issue, our group established a reliable framework to detect bubbles in different types of bubbly flows with volume void fractions as high as 2% by rigorously synthesizing digital image processing algorithms^5,8^; however, the limitation of this approach still exists. Most importantly, conventional methods require the optimization of coefficients or thresholds by trial-and-error, and they are not universally applicable to various types of bubbly flow. Therefore, detection accuracy largely varies depending on the skillfulness of the researcher, and the overall processing cost including human resources is very high.

In recent years, deep learning has been recognized as a powerful tool in the field of digital image processing, and has also proved promising in addressing various problems in fluid mechanics^20–25^. These studies are finding ways to overcome long-lasting problems by applying a deep learning-based methodology to solve governing equations or to improve experimental techniques, which have been shown to enhance model accuracy and save on overall data processing cost, which is dominated by human resources. Recent experiments in multiphase flow studies attempted to detect objects (e.g., bubbles, droplets, and particles) by combining deep learning models like object detection models such as Faster R-CNN^26^ with conventional image processing in gas–liquid two-phase flow^27–33^. Cerqueira and Paladino^27^ determined the best fitted ellipse of each candidate bubble using the region proposal algorithm and a CNN (convolution neural network), and Poletaev et al.^31^ found the center, axes, and orientation of each bubble in a bubbly jet flow using an autoencoder and a CNN classifier. To understand the detailed interactions between each phase, however, it is important to know the exact shape (not just the bounding box or fitted ellipse) of the gas–liquid interface, which has not been possible previously. The aforementioned studies have a distinct limitation in that it is not feasible to obtain the actual bubble geometry under the shape instability (i.e., wobbling or deformation) caused by various flow conditions, because they considered a narrow range of bubble shapes, namely spherical or synthetic (artificially manipulated). It should also be noted that testing (validation) of the trained model with untrained data is missing in most previous studies.

Therefore, in the present study, we develop and validate a fully automated tool to detect and extract the actual shape of bubbles based on a deep-learning framework, which can be universally applied to various types of two-phase flows. We focused on instance segmentation, which extracts a pixel-wise segmentation mask of each detected instance, one of the representative challenges in the area of computer vision^34,35^. It has been actively adopted in fields where it is necessary to identify each instance under harsh conditions, such as high noise or variation in image contrast and color. For example, there are many studies in biology and biotechnology that need to identify each cell or tissue in a complex image^36–39^. We train the Mask R-CNN^34^, one of the instance segmentation models, with training data composed of bubbly flow data obtained experimentally under different conditions and synthetic bubble images. We optimized the amount and composition of training data from different sources and used a variety of image augmentation methods to optimize model performance. Typically, the object detection model requires a large amount of training data, but we were able to achieve a high detection performance with a relatively small but rigorously refined dataset. In addition, a customized loss function was used to improve the performance of small bubble detections, which is typically poorer than that of larger object detections^34,36^. As a result, we obtained a fully automated bubble detection and mask extraction tool that is effective in different gas–liquid bubbly flows without manually tuning the thresholds. We hope this will be useful in reducing the difficulties in the analysis of the optical images of multiple objects interacting in a complex manner.

## Training and evaluation of the algorithm

### Data acquisition and optimization

**Figure 1:**
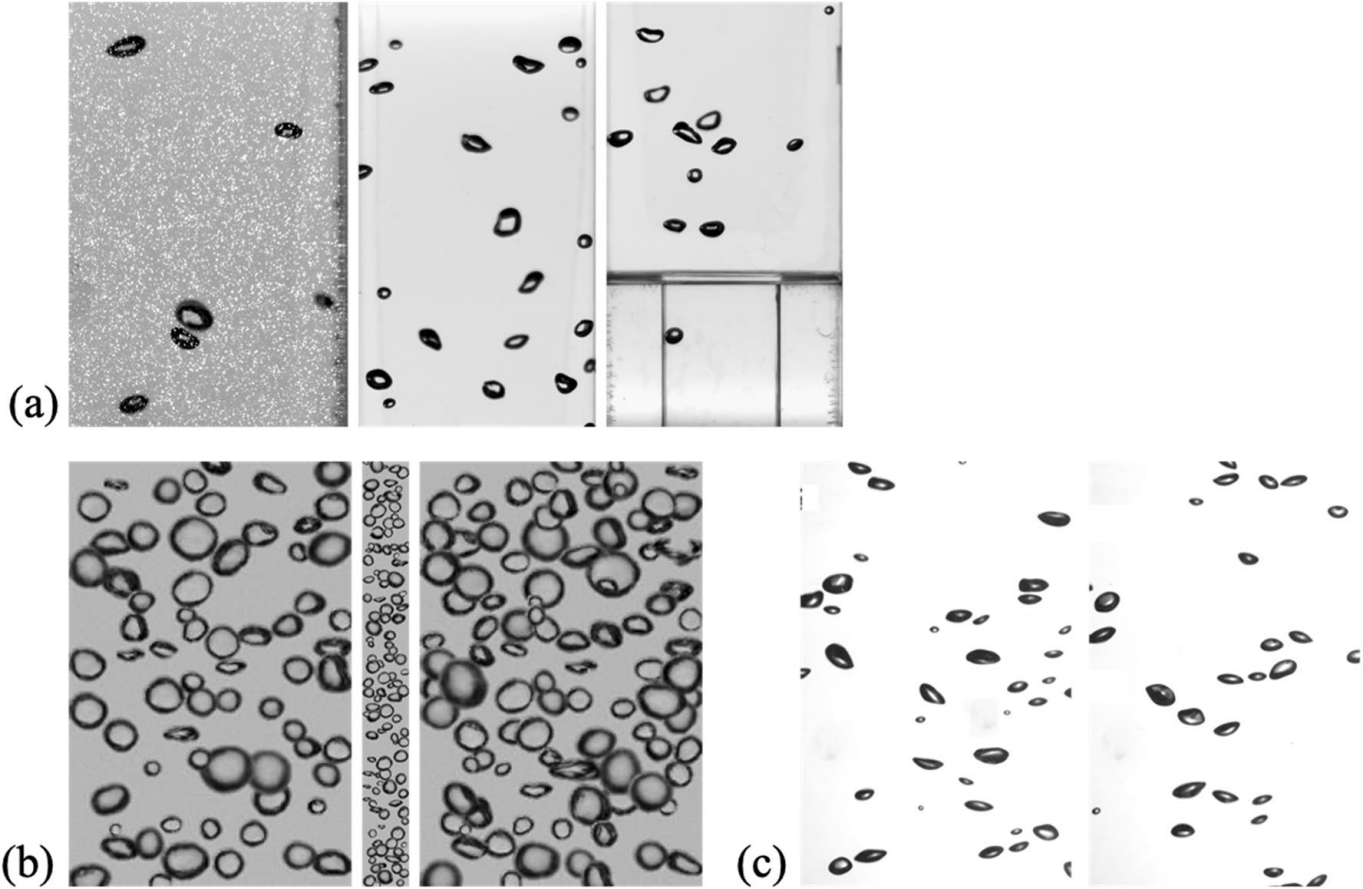
Examples of bubbly flow images used as a training and test dataset: (**a**) experimental data of upward bubbly flows in an expansion pipe^8^; (**b**) synthetic bubble images from BubGAN^40^; (**c**) experimental data of bubble-swarm flow^9^.

For a training dataset, we used both experimental and synthetic bubbly flow images obtained from the upward bubbly flows in an expansion pipe^8^ and BubGAN algorithm^40^, respectively (Fig. 1a,b). The experimental bubbly flow data included bubbles with a size range of 7–98 pixels (0.25–3.4 mm in physical scale), and its volume void fraction was 0.72%. As shown in Fig. 1(a), they were obtained using two different techniques: two-phase particle image velocimetry (PIV) and shadowgraph. Whereas the shadowgraph visualizes the bubble shadow only, the two-phase PIV measures the liquid-phase velocity as well as the bubble statistics (shadows). Thus, the images obtained using the two-phase PIV were added to the training dataset to make the model robust in environments, wherein the optical image has a significant level of noise (represented by seeding particle images). In addition, the training dataset would benefit from the fact that the distribution of gray levels in the image differs depending on the optical setup^8,9^. Because one of our primary goals is to improve the performance of disassembling of overlapped bubbles, the conditions possibly missing in the experimental dataset can be supplemented by the synthetic dataset in which the size and distribution of bubbles are controlled. For the data produced by the BubGAN, the bubble size was varied between 4 and 123 pixels, and the intersection over union (IoU) between two bounding boxes (of each bubble) was set as IoU_B_ = 0.11, 0.16, and 0.2 (Fig. 1(b)). A much higher value of IoU_B_ caused negative effects, in that the dense bubble population would lead to excessive split of bubbles. Here, the IoU indicates the ratio of the overlapping area between two objects to the union area. The void fraction of the synthetic dataset was set to 3.0–8.0%. To add bubbles smaller than the average size of 35 pixels in diameter, on the other hand, the height of the image with IoU_B_ = 0.16 was adjusted to three times longer than for other cases (IoU_B_ = 0.11 and 0.2), because all training inputs are scaled to be the same size (640 × 640 pixels), regardless of the physical size of the image.

Although some of the images from the same experimental conditions as the training dataset were used to evaluate the model, we also added the experimental data of bubble-swarm flow^9^ to the test dataset, which was not included in the training set (Fig. 1(c)). The bubble size range in the bubble-swarm flow data in the test set was 7–65 pixels (0.6–5.2 mm) with a volume void fraction of 0.3–2.0%. For all experimental images in the training and test datasets, the overlapped bubbles that were difficult to obtain the exact separated mask (ground truth) were removed to avoid detrimental effects to the model. The conditions of the training and test datasets are listed in Table 1.

**Table 1:** Details for training and test datasets.

Source of data		Bubbly flow in an expansion pipe^8^		BubGAN^40^	Bubble-swarm flow^9^
		two-phase PIV	Shadowgraph		
Resolution [pixels]		736 × 1120	576 × 1032	200 × 300 100 × 900	624 × 976
Bubble size [pixels]		7–98 (46.8)	10–60 (31.4)	4–123 (34.0)	7–65 (28.3)
Volume void fraction [%]		0.72	0.72	3.0–8.0	0.3–2.0
Training set	# of images	1588	854	150	–
	# of bubbles	8160	14,640	15,280	–
Test set	# of images	8	8	8	8
	# of bubbles	40	120	770	330

The numbers in the bracket denote the averaged bubble size.

In general, a model trained with more data performs better, but there is a practical limit to the amount, as well as the level of quality, of data that can be obtained from the experiments. Therefore, we needed to optimize the composition of the training dataset and ran several experiments to determine the optimal condition. In other words, two models were trained for the same iterations, the first one was trained with the experimental data, whereas the second one was trained with the synthetic data. They were then evaluated with the same test dataset: 30% from the upward bubbly flow in an expansion pipe, 45% from the synthetic bubble images, and 25% from the bubble-swarm flow. The model trained with the synthetic data only exhibited half the accuracy (AP_50_, average precision (AP) for the cases of IoU ≥ 0.5) as the model trained only with the experimental data, thereby indicating that the model trained with only the synthetic dataset (even though its size is large) does not provide the desired performance. The experimental data play a critical role in transferring the ability to recognize the actual bubble shapes under various conditions.

By adding synthetic bubble images to the training set of the experimental data, it was enhanced to disassemble the overlapped bubbles. Moreover, we found that the accuracy (AP_50_) increased slightly if the training dataset included the experimental images without a brightness gradient inside the bubble shadows, i.e., if all the bubbles were completely filled with black color. The optimized compositions of the training and test datasets are presented in Table 1.

### Deep learning model and training configurations

Mask R-CNN is an instance segmentation model that labels each pixel corresponding to each instance detected by adding a parallel mask branch to Faster R-CNN, a widely used object detection model. In this study, we used the Matterport Mask R-CNN implementation (https://github.com/matterport/Mask_RCNN), using ResNet-101 as the backbone and applied transfer learning from pre-trained COCO weights (https://github.com/matterport/Mask_RCNN/releases/download/v2.0/mask_rcnn_coco.h5) to maximize the data efficiency and delay overfitting. The model was trained for 24 epochs using a batch size of 1, with an initial learning rate of 10^–4^, which was optimized for our computing environment using a grid search (from 10^–2^ to 10^–5^), while decreasing it by a factor of 10 after every 10 epochs. From the entire model, only ResNet stage 5 and the head layer were re-trained, and it was empirically shown that the highest accuracy was achieved before the occurrence of overfitting, compared to the selection of other layers to be trained. In addition, to slow down the overfitting, we applied several image augmentations, such as flipping, rotation, and Gaussian noise addition, randomly to the training input image at each iteration. For the training, ADAM was chosen as an optimizer, and the regularization weight decay value was set to 10^–4^. The training was conducted on a single NVIDIA RTX 2080 Ti GPU.

In general, large objects in an image have a dominant influence on the training loss of the object detection model^34,36^, and thus the accuracy of smaller objects detection tends to be low. Regarding the bubble detection problem, however, the detection accuracy of small bubbles is as important as that of large bubbles, because the bubble size follows a Gaussian distribution in a typical gas–liquid two-phase flows, and their scale-wise interactions are especially important in studying the transport phenomenon^5,8,9^. Therefore, we improved the mask accuracy of small bubbles using a customized loss function that increases or reduces the effect of the bubble size on the loss by weighting the loss according to the bubble size (more details are provided in the Methods section). As a result, the mask accuracy of small bubbles (AP_*S*_) increased by approximately 4%, and the overall accuracy (AP_50_) slightly increased (the definitions of AP_x_ are provided in the next section). More configuration details can be found in our code, which is available online (https://github.com/ywflow/BubMask).

### Evaluation of the bubble mask extraction performance

The performance of the model was evaluated by calculating the mask AP for each mask IoU threshold and object size range, following the COCO evaluation metrics (https://cocodataset.org/#detection-eval). The evaluation metrics used include the AP (averaged over IoU thresholds from 0.5 to 0.95 with intervals of 0.05), AP_50_ (for IoU ≥ 0.5), and AP_75_ (IoU ≥ 0.75) according to the IoU threshold, and AP_*S*_, AP_*M*_, and AP_*L*_ according to the bubble size of the test dataset. Here, the subscript refers to the IoU threshold as a percentage or the size range of the bubble. The ranges of bubble size (d_b_) for the AP_*S*_, AP_*M*_, and AP_*L*_ were determined by classifying all bubbles in the test dataset into small (d_b_ < 22.6 pixels), medium (22.6 pixels ≤ d_b_ < 39.5 pixels), and large (d_b_ > 39.5 pixels), respectively, and they cover 36%, 38%, and 26% of the total number of bubbles tested. This ratio was determined intentionally to evaluate the effect of the customized loss function on the model performance quantitatively, especially for detecting small bubbles. It is noted that each type of AP for each image was averaged over all corresponding images in the test dataset, and not just from a single test, while maintaining the same number of images for all types of data. This is because the accuracy of each image is also important to confirm the universality of the present model which works in various complex two-phase flows; as mentioned above, each test image has different levels of bubble density, bubble locations, image background, and lighting conditions, which requires the development of a universal model.

## Results and discussion

### Bubble detection and mask extraction

**Figure 2:**
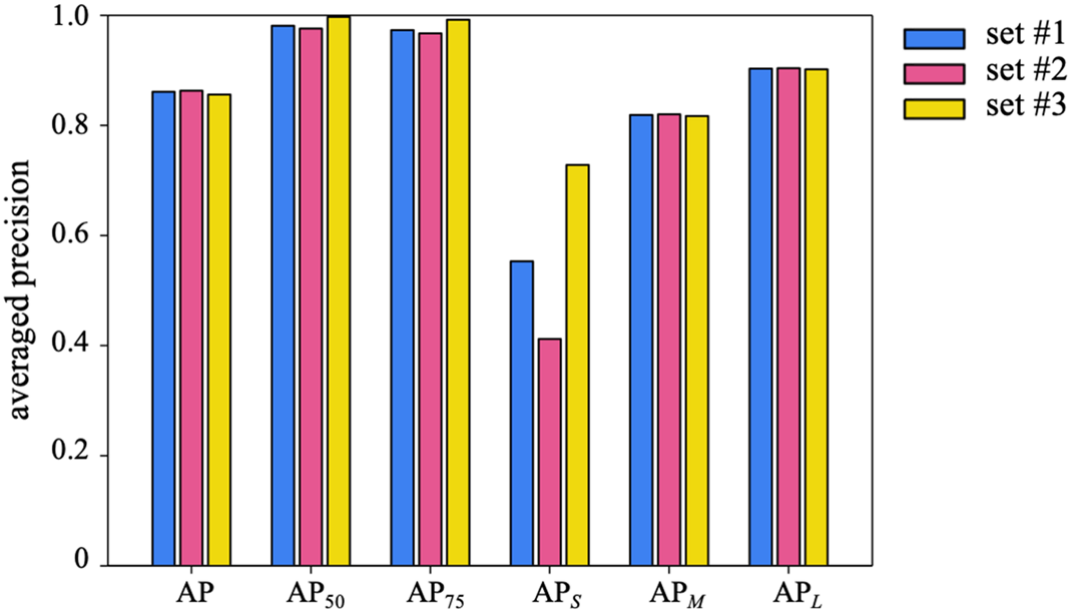
Mask averaged precision based on the IoU threshold and object size range. set #1: all test dataset in Table 1, set #2: images with similar experimental conditions to the training set^8,40^, set #3: images with different experimental conditions from the training dataset^9^.

Figure 2 shows the accuracy (AP) of the present model depending on the IoU threshold value and object size range, evaluated using three test datasets, which are designated as set #1, #2, and #3, respectively. Each test set included all the test images, images from similar experimental conditions to the training set^8,40^, and images of different experimental conditions^9^ from the training dataset (see Table 1). The present model exhibits a high accuracy not only for test set #2, which has similar experimental conditions as the training set, but also for set #3, which was not included in the training set. While the accuracy variation among the test sets is not substantial in general, the difference in AP_*S*_ between test sets #2 and #3 is relatively large. This is because the bubbles in the small size range of set #2 are smaller than the bubbles in the small size range of set #3. Even if the magnitude of the mask difference between the ground truth and the detected mask is the same for the large bubbles, the IoU is largely reduced for small bubbles, resulting in a significant decrease in the accuracy of detection.

**Figure 3:**
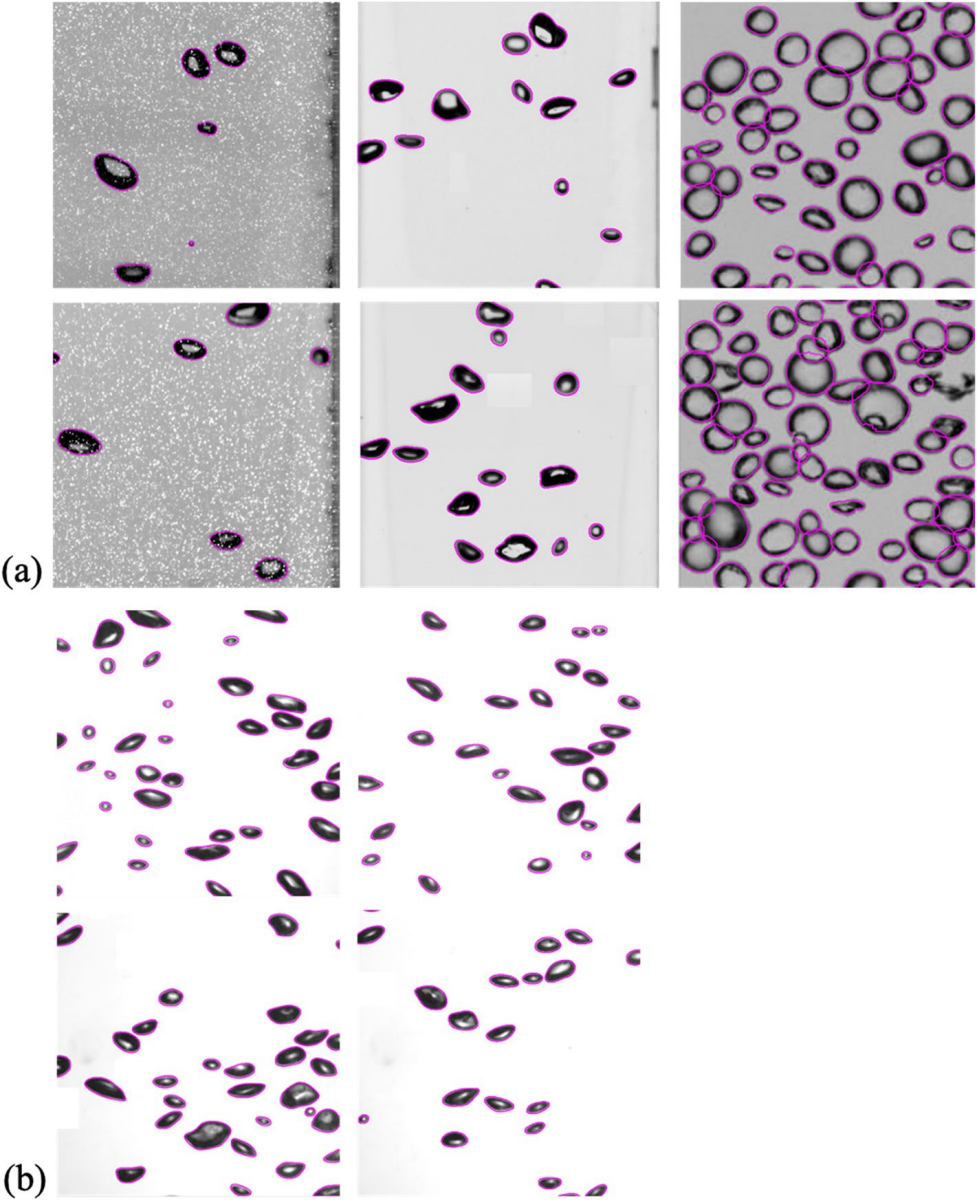
Bubble detection examples (IoU threshold of 0.5) for (**a**) test set #2 and (**b**) #3. Here, the purple solid lines denote the extracted bubble shapes, and the images were cropped from original images and scaled for better visibility.

The representative results of bubble edge (mask) detection by the present model (for an IoU threshold of 0.5) are shown in Fig. 3. It is clear that the detected bubble shapes follow the actual bubble shadows quite well. Based on the results shown in Figs. 2 and 3, we deem it reasonable to represent the performance of the present model based on AP_50_, because the difference between AP_50_ and AP_75_ is small, and the IoU between the ground truth and predicted mask would increase due to human error relating to the process of labeling the ground truth mask (bubble edge). The AP_50_ for the entire test dataset (set #1) of the present model is 0.981 (it is 0.997 for set #3), which is a promising bubble edge detection performance.

### Assessment of model performance depending on bubbly flow conditions

**Figure 4:**
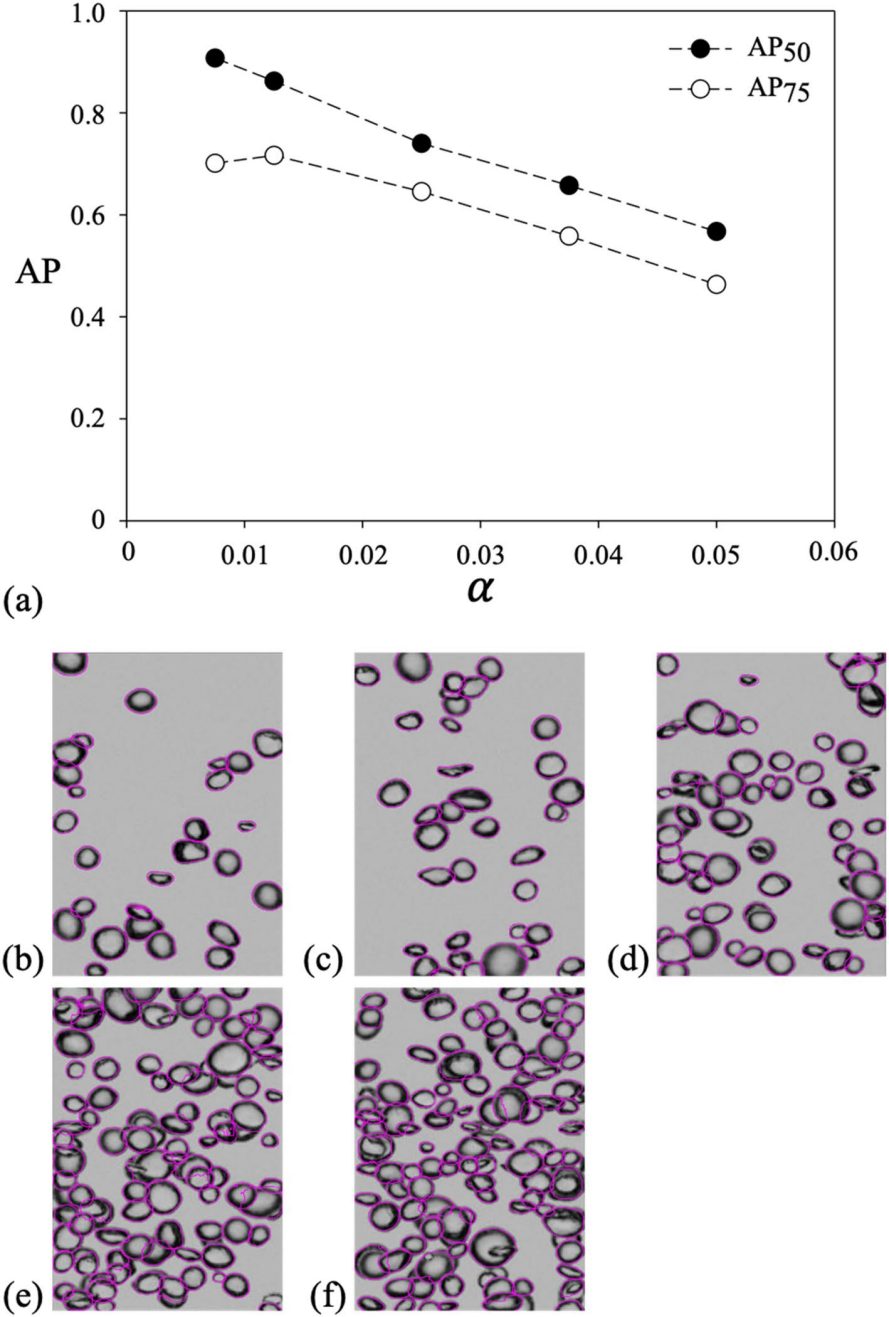
(**a**) Variation of AP_50_ and AP_75_ of synthetic images with volume void fraction (α). Representative bubble images with detection results (purple solid lines) are shown for α of (**b**) 0.0075; (**c**) 0.0125; (**d**) 0.0250; (**e**) 0.0375; (**f**) 0.0500.

In this section, we present the assessment of the present model’s performance in several ways to confirm its effectiveness under a wide range of experimental and/or flow conditions. First, we analyzed the dependency on the volume void fraction (*α*) of the model performance, which is one of the most important parameters for characterizing the physics of bubbly flows. In Fig. 4(a), we plotted the variation of AP_50_ and AP_75_ depending on the void fraction. Because ground truth (separated bubble edges) for overlapped bubbles are required for a fair evaluation, the evaluation was performed using the synthetic bubble images. The results of 50 synthetic images were averaged for each void fraction. As expected, the accuracy tended to decrease as the void fraction increased, and AP_50_ and AP_75_ reached 0.567 and 0.463, respectively, when the void fraction increased to 5%. Considering that the typical maximum void fraction in the experimental studies on bubbly flows using optical measurements is approximately 2–3% (mostly below 1%)^5,41,42^, the AP_50_ is higher than 0.71–0.8 (0.9 for void fraction below 1%), which is acceptable. Some representative result images for each void fraction are presented in Fig. 4(b)–(f), which qualitatively demonstrate the operating range and performance of the present model.

**Figure 5:**
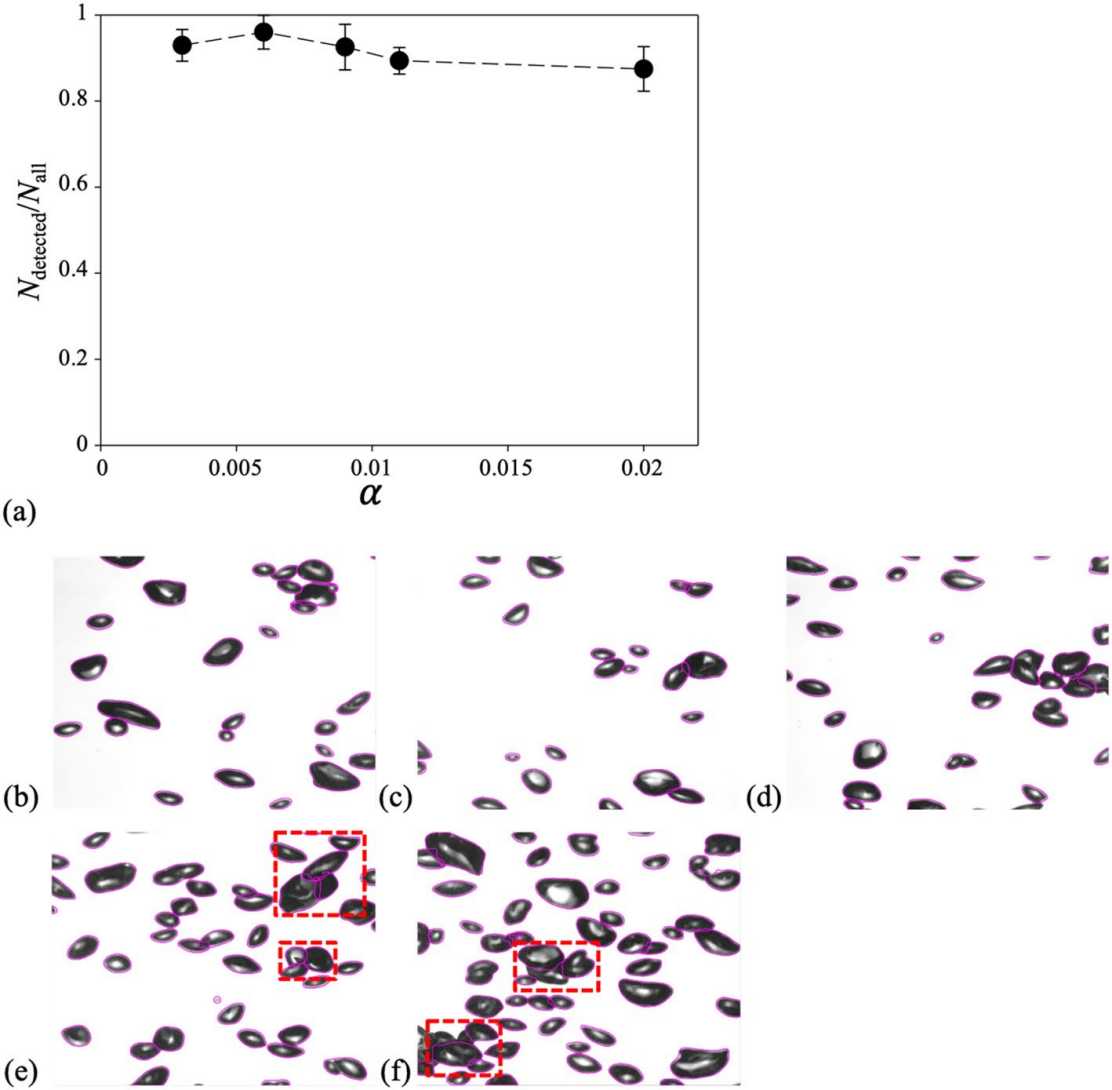
(**a**) Ratio of the number of detected bubbles (*N*_detected_) to the total number of bubbles in the bubble-swarm flow images^9^ with volume void fraction (α). Representative bubble images with detection results (purple solid lines) are shown for α of (**b**) 0.003; (**c**) 0.006; (**d**) 0.009; (**e**) 0.011; (**f**) 0.02.

Next, we test the model with experimental bubble-swarm flow data^9^, which includes a larger number of overlapped bubbles than the images used in the training dataset, to determine the effectiveness of the present model in two-phase flows with a moderate void fraction (up to approximately 2%). Unlike that for synthetic images, obtaining the exact individual shape from all the overlapped bubbles is not feasible in this case; thus, the ratio of the number of bubbles detected by the model to the total number of bubbles was calculated depending on the volume void fraction (Fig. 5(a)). Here, the results of 10 images were averaged for each corresponding void fraction, and the representative result images were also presented to judge the operating range and performance of the present model qualitatively (Fig. 5(b)–(f)). More than 92% of the bubbles were detected for void fractions of up to 1%. The loss of detection slowly increased with the void fraction; however, more than 87% of the bubbles were detected (within 5% standard deviation), even for an intermediate void fraction of 2%. As shown in Fig. 5(f), the bubbles are severely overlapped, even with a void fraction of 2%. The capability of the present model to identify individual bubbles with corresponding masks among the overlapped bubbles is well demonstrated in bubble clusters without a clear bright core (highlighted with dashed boxes in Fig. 5(e,f)). It is noted that some image processing algorithms, as discussed previously, use the bright spot inside the bubble shadow to distinguish an individual bubble from the cluster.

**Figure 6:**
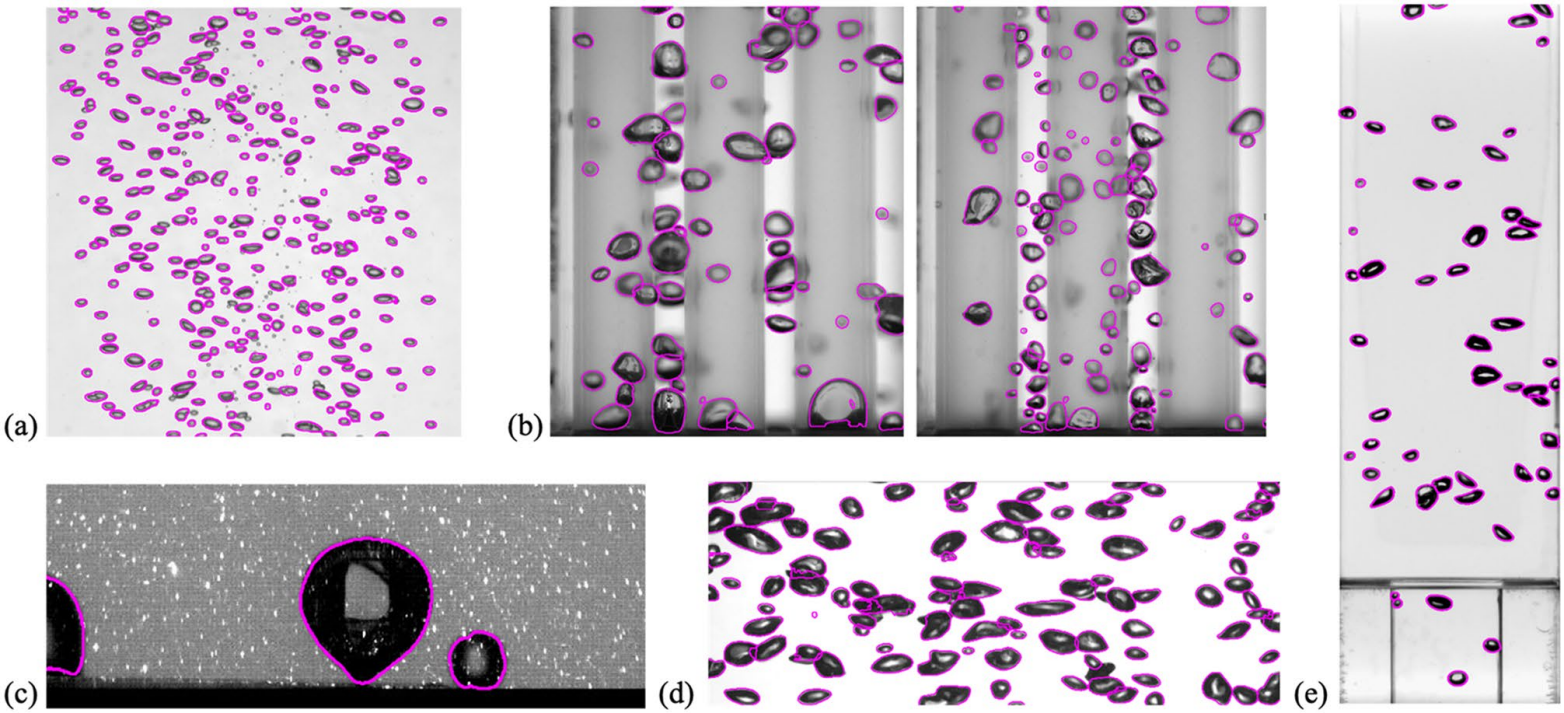
Bubble detection and mask extraction results for various gas–liquid two-phase flow experiments: (**a**) bubble plume (*J*_*G*_ = 0.033 × 10^–3^ m/s, *d*_e_ = 1.8 mm) (unpublished data); (**b**) bubbly flow in a rod-bundle of nuclear power plant (*J*_*G*_ = 0.008 m/s, *J*_*L*_ = 0, 0.68 m/s, *d*_e_ = 2.8 and 2.0 mm) (unpublished data); (**c**) pool boiling bubble (*T*_*w*_ = 104 ℃, *T*_*l*_ = 98 ℃, *d*_e_ = 3.1 mm) (unpublished data); (**d**) bubble-swarm flow^9^ (α = 0.021, *d*_e_ = 4.2 mm); (**e**) upward bubbly flow in an expansion pipe^8^ (α = 0.0072, *d*_e_ = 3.0 mm). *J*_*G*_: gas flow rate, *J*_*L*_: liquid flow rate, *d*_e_: mean bubble equivalent diameter, *T*_*w*_: wall temperature, *T*_*l*_: liquid temperature, α: volume void fraction.

Finally, we have shown the results of bubble detection and mask extraction achieved by the present model for different types of gas–liquid two-phase flows (Fig. 6), of which the visualization data came from our group (published and unpublished data). The tested two-phase flow includes the bubble plume (Fig. 6(a), unpublished), bubbly flow in a rod-bundle geometry in a nuclear power plant (Fig. 6(b), unpublished), pool boiling bubble (Fig. 6(c), unpublished), bubble-swarm flow (Fig. 6(d), Lee and Park^9^), and upward bubbly flow in an expansion pipe (Fig. 6(e), Kim and Park^8^). It should be noted that these data were not included in both the training and test dataset. As shown, it was qualitatively demonstrated that the present model can be universally applied to diverse two-phase flows, for the purpose of detecting and extracting an individual bubble. It is also promising to see that the bubbles in the interaction with the solid wall, such as adhesion, bouncing, and sliding, can also be detected (Fig. 6(b,c,e)). In the supplementary video, we further demonstrate how the extraction of exact bubble shapes can be used to track individual bubbles in the spatiotemporally varying bubbly flows.

### Saving on mask extraction time

**Figure 7:**
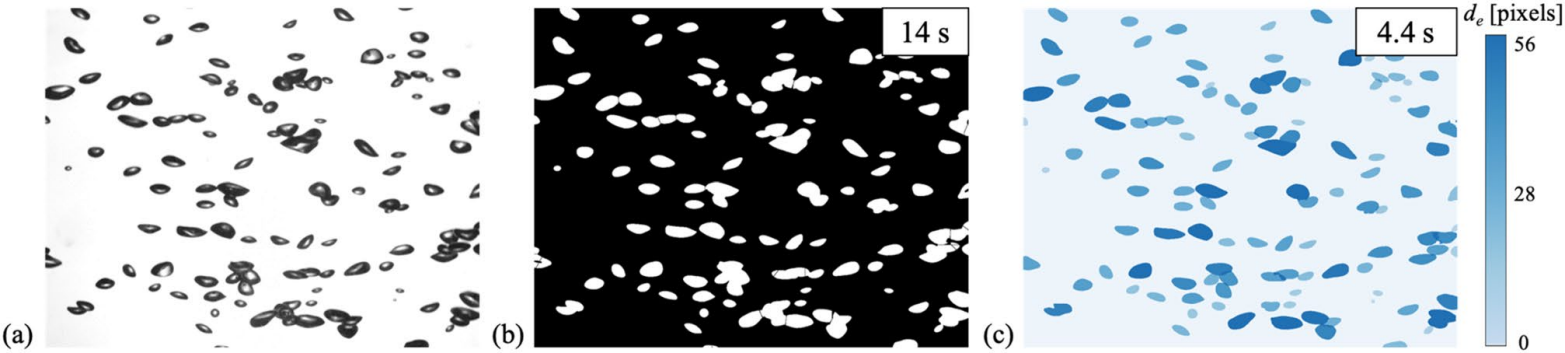
Example of the comparison of mask extraction and corresponding time consumption: (**a**) raw image; (**b**) result of conventional image processing; (**c**) result of present model. The image was obtained from the bubble-swarm flow^9^ with void fraction of 0.9% and has a resolution of 1248 × 976 pixels.

When processing the optically obtained experimental data, computational speed is also an important issue as its accuracy. Because the conventional multiple-filter image processing technique is now being replaced with convolutional layers in the present model, it is expected that the time required for mask extraction would be reduced. When we use the same computing resources to test the same images, the calculation time of the present model is two to three times shorter than that of the conventional method. It should be noted that the bubble mask extracted by the present model is at least equivalent to or better than the results from conventional image processing (Fig. 6). Figure 7 shows an example of the extraction of bubble masks and a time cost comparison between the present model and the conventional method. Here, the bubble-swarm flow^9^ of 0.9% void fraction was compared, and the Watershed transform was applied twice repeatedly as a conventional method. As shown, the extracted bubble masks are equivalent to each other, but the time cost (averaged for 10 images) significantly decreased to 4.4 s from 14 s taken by the Watershed transform.

## Concluding remarks

In the present study, we successfully developed a fully automated and universal bubble detection and shape extraction tool by training the Mask R-CNN with an optimized dataset including experimental images of bubbly flows and realistic synthetic bubble images (produced by BubGAN). We customized the loss function to weight the effect of bubble size on the detection performance and enhanced the accuracy of detecting small bubbles (AP_S_) by 4%, thereby improving overall accuracy as well. The present model shows good universality under a wide range of experimental conditions and high detection performance owing to dataset optimization and a unique loss weighting system according to bubble size, which was possible based on the physical understanding of bubbly flows. We also applied randomized data augmentation such as adding noise and generating black bubbles, which helped to improve accuracy, as in other studies. As a result, the averaged precision (AP_50_) for the entire test dataset (which includes the bubble-swarm flow data not included in the training set) of the present model reached 0.981. Furthermore, we confirmed that the present model works well on a variety of experimental (optical setup) and flow conditions, even if the data were not included in the training dataset. Finally, the mask extraction time was significantly reduced compared to that of the conventional image processing method. What is remarkable here is that the present model no longer requires human intervention (trial-and-error) during the mask extraction process, thus reducing the overall processing cost. Based on our experience while training and testing the model, to improve the performance of the model, we suggest resizing the image to different scales and merging the obtained masks of multiple images adjusted to different scales.

In a future work, we plan to further improve the model by combining other deep-learning-based algorithms (e.g., deep learning optical flow) and using other bubble image features such that it can be readily applied to much wider experimental conditions (or harsh conditions in terms of optical configuration), such as low contrast images, multiple fluid layers with different refractive indices, and severely high void fraction flows. Although the present model was developed focusing on gas–liquid two-phase flows, we believe that it can be extended to other areas where the separation of objects in optically visualized images is required, as in studies on droplet (particle)-laden flows.

## Methods

### Preparing data for training

For the training and evaluation of the model, we need raw images of bubbles with ground truth masks for each bubble. We followed the conventional image processing method for optical gas–liquid two-phase flow experiments that our group has established^5,8,9^ to create the ground truth mask of the experimental images. First, the images were binarized using a median filter and Sauvola binarization^43^. Then, the bright bubble core was filled using the morphological image reconstruction algorithm^44^ and denoised using a size filter^5^. Next, each object in the binarized image was identified to determine whether it was an overlapped bubble cluster or solitary bubble using a roundness criterion^14^ based on the relationship between the perimeter and area of the bubble. After the overlapped bubble clusters were identified, they were removed from both the raw and binarized images using an in-house MATLAB code. If any overlapped bubble cluster that was indistinguishable by a roundness criterion still remained, it was also removed manually using the MATLAB GUI tool. As a result, we obtained bubble images with only solitary bubbles and binary masks for each bubble in the image.

### Weight for the loss function

To increase the model accuracy for small bubbles as much as that for large bubbles, we apply the weight factor to the loss function to increase the contribution of small bubbles to the training losses (smooth L1 loss). The customized weights are given by Eq. (1), where *size* denotes the bubble equivalent diameter (d_b_), and *w* is the weight effect factor, which is 0.3 in the present study.

1 $$ Global\;weight = \left( {\frac{{size^{ - 1} - size_{\max }^{ - 1} }}{{size_{\min }^{ - 1} - size_{\max }^{ - 1} }} - 0.5 } \right)w + 1 $$

To apply the global weights to the loss function rather than the local weights, which only work on each iteration (image), the minimum and maximum bubble sizes of all the bubbles in the training set are used. We have empirically found that weighting only small bubbles is more effective than weighting small and large bubbles.

## Supplementary Information

Supplementary Video 1.

Supplementary Information 1.
