# Supplementary material for: Deep learning-based automated and universal bubble detection and mask extraction in complex two-phase flows
Source: Sci Rep. 2021 Apr 26;11:8940. doi: 10.1038/s41598-021-88334-0 (PMC8076184; doi:10.1038/s41598-021-88334-0)
Supplement: Supplementary file 2 — Supplementary Information 1. [file 41598_2021_88334_MOESM2_ESM.docx]

**Supplementary Material for**

Deep learning-based automated and universal bubble detection and mask extraction in complex two-phase flows

Yewon Kim^1^ and Hyungmin Park^1,2,*^

^1^Department of Mechanical Engineering, Seoul National University, Seoul 08826, Korea

^2^Institute of Advanced Machines and Design, Seoul National University, Seoul 08826, Korea

^*^Author to whom correspondence should be addressed: hminpark@snu.ac.kr

Movie. Results of detection and extraction masks of complex bubbly flows: (left) bubbly flow in an expansion pipe: volume void fraction of 0.72% and mean bubble size of 3.0 mm; (right) bubbly flow in a rod-bundle geometry: gas flow rate of 0.008 m/s, liquid flow rate of 0.45 m/s and mean bubble size of 2.3 mm.
